# A distributed penalty-based zeroing neural network for time-varying optimization with both equality and inequality constraints and its application to cooperative control of redundant robot manipulators

**DOI:** 10.3389/fnbot.2025.1553623

**Published:** 2025-03-17

**Authors:** Liu He, Hui Cheng, Yunong Zhang

**Affiliations:** ^1^School of Computer Science and Engineering, Sun Yat-sen University, Guangzhou, China; ^2^School of Intelligent Systems and Engineering, Sun Yat-sen University, Shenzhen, China

**Keywords:** distributed optimization, zeroing neural network, equality and inequality constraints, time-varying, cooperative control

## Abstract

This study addresses the distributed optimization problem with time-varying objective functions and time-varying constraints in a multi-agent system (MAS). To tackle the distributed time-varying constrained optimization (DTVCO) problem, each agent in the MAS communicates with its neighbors while relying solely on local information, such as its own objective function and constraints, to compute the optimal solution. We propose a novel penalty-based zeroing neural network (PB-ZNN) to solve the continuous-time DTVCO (CTDTVCO) problem. The PB-ZNN model incorporates two penalty functions: The first penalizes agents for deviating from the states of their neighbors, driving all agents to reach a consensus, and the second penalizes agents for falling outside the feasible range, ensuring that the solutions of all agents remain within the constraints. The PB-ZNN model solves the CTDTVCO problem in a semi-centralized manner, where information exchange between agents is distributed, but computation is centralized. Building on the semi-centralized PB-ZNN model, we adopt the Euler formula to develop a distributed PB-ZNN (DPB-ZNN) algorithm for solving the discrete-time DTVCO (DTDTVCO) problem in a fully distributed manner. We present and prove the convergence theorems of the proposed PB-ZNN model and DPB-ZNN algorithm. The efficacy and accuracy of the DPB-ZNN algorithm are illustrated through numerical examples, including a simulation experiment applying the algorithm to the cooperative control of redundant manipulators.

## 1 Introduction

Recently, owing to the development of large-scale networks and advancements in big data theory, research on distributed optimization problems has garnered considerable attention due to its broad application prospects in science and engineering (Bahman and Jorge, 2014; Molzahn et al., 2017; Mao et al., 2023; Kang and Yang, 2023), such as resource allocation (Cai et al., 2024), energy management (Li et al., 2024), and economic dispatch (Huang et al., 2022). In a typical distributed optimization problem, the objective function of a multi-agent system (MAS) results from summing the local sub-objectives of each individual agent (Jia et al., 2024). In addition, during the search for an optimal solution, each agent only has access to its local sub-objective and constraints while communicating its own state to neighboring agents. Overall system convergence is achieved through consensus among the agents.

The aforementioned studies focus on distributed optimization problems with static objective functions. However, distributed time-varying constrained optimization (DTVCO) problems, where the objective functions and constraints change over time, are encountered in practical scenarios, such as the formation control of multi-robot systems (Sun et al., 2022), Nash equilibrium seeking for time-varying non-cooperative games (Ye and Hu, 2015), and distributed time-varying resource allocation (Cherukuri and Cortés, 2016). The DTVCO problem is more intricate than distributed static optimization since the optimal solution evolves over time. As a result, researchers have increasingly turned their attention to DTVCO (Zheng et al., 2017; Sun et al., 2023; Zhang et al., 2021a; Zhu and Wang, 2024; He et al., 2021). For example, Zheng et al. (2017) introduced a consensus-based control scheme employing second-order optimization methods to address DTVCO problems characterized by local objective functions with identical Hessians. Sun et al. (2023), introduced a distributed control algorithm comprising a sliding-mode consensus component and a Hessian-based optimization component, integrated with log-barrier penalty functions, to address the DTVCO problem. Zhu and Wang (2024) utilized the finite-time stability theory and graph theory to propose a novel class of distributed finite-time optimization algorithms that are independent to time derivatives of the gradients and Hessian information.

Due to the parallel computing characteristics, hardware implementation capabilities, and parallel distributed properties (Yang et al., 2020; Jin et al., 2022b; Qiu et al., 2021; Sun et al., 2020; Song et al., 2024), neural network-based methods provide an powerful approach for solving various challenging computational problems in real time. Recent advancements in neural networks have shown significant progress in tackling time-varying problems. For fault-tolerant motion planning of redundant manipulators, Jin et al. (2017) developed a different-level simultaneous minimization scheme by utilizing a discrete-time recurrent neural network to solve quadratic programming problems. Khan et al. (2020b) have developed a metaheuristic-based control framework for simultaneous tracking control and obstacle avoidance of redundant manipulators, unifying these tasks into a single constrained optimization problem. Enhancements to the beetle antennae search algorithm, called BAS-ADAM, have been proposed by Khan et al. (2020a) to improve convergence behavior and avoid local minima in highly non-convex objective functions. A distributed competitive and collaborative coordination approach has been established by (Liu et al., 2024) for multi-robot systems, optimizing system stability and resource utilization through a fusion of recurrent neural dynamics and distributed solvers. In addition, Zhang et al. (2018a) have proposed a novel recurrent neural network for kinematic control of redundant manipulators, addressing periodic input disturbances and physical constraints while optimizing a general quadratic performance index. These studies collectively highlight the innovation and potential of neural networks in addressing time-varying challenges in various applications. Another exciting direction in neural network-based control scheme is the data-driven model predictive control (MPC). The data-driven MPC impacted the field of robotic control, particularly in handling complex and dynamic environments. For example, one study by Yan et al. (2024) proposed a data-driven MPC algorithm that integrates neural dynamics for trajectory tracking in redundant manipulators with unknown models. Another research by Jin et al. (2024) introduced a cerebellum-inspired learning and control scheme using echo state networks to achieve precise joint velocity control in redundant manipulators.

Introduced by Zhang et al. (2002), since its inception, zeroing neural network (ZNN) method has been widely utilized to address a range of time-varying problems (Liao et al., 2022; Peng et al., 2022; Lan et al., 2023; Zuo et al., 2022; Dai et al., 2023; Chen et al., 2024; Hu et al., 2024; Jin et al., 2022a; Tan et al., 2023; Ding et al., 2018), such as time-varying matrix inversion (Dai et al., 2023), time-varying optimization (Chen et al., 2024), and time-varying Lyapunov equation solving (Zuo et al., 2022). Recent advancements in ZNN have illustrated their effectiveness in various robotic applications. (Sun et al., 2024b) have successfully applied ZNN to human-robot interaction and force control, improving estimation accuracy and ensuring safety in complex environments. In the realm of motion estimation, (Wang et al., 2024) have integrated ZNN into a multi-task parallel learning framework to reconstruct missing sEMG signals and estimate joint angles with high accuracy. In addition, (Xie et al., 2025) have utilized ZNN in obstacle avoidance schemes for redundant robots, leveraging deep reinforcement learning to enhance obstacle avoidance capabilities. Furthermore, Sun et al. (2024a) have developed a hybrid orthogonal repetitive motion and obstacle avoidance scheme for omnidirectional mobile robotic arms, achieving accurate obstacle avoidance and repetitive motion tasks. These studies collectively highlight the innovation and potential of ZNN in revolutionizing various aspects of robotics.

Despite the significant success of ZNN method in addressing time-varying problems, the ZNN models presented in the aforementioned works are limited to solve those problems in a centralized manner. There is a scarcity of existing research focusing on distributed ZNN. The most typical work of distributed ZNN is by Jin et al. In Jin et al. (2018), a ZNN-based control scheme is introduced to address the cooperative motion generation problem. This scheme facilitates cooperative motion generation within a distributed network of multiple redundant manipulators. However, for an individual manipulator, the computation results of neighbors' control inputs are required to compute its own control input. Therefore, the proposed control method is not a fully distributed scheme, as the control inputs for the entire system need to be computed collectively by all manipulators, rather than being computed independently by each manipulator.

Driven by the above discussions, a novel penalty-based ZNN (PB-ZNN) model is proposed. The PB-ZNN model solves a continuous-time DTVCO (CTDTVCO) problem in a semi-centralized manner. Then, to extend the application of ZNN models to distributed time-varying problems, a distributed penalty-based ZNN (DPB-ZNN) algorithm is designed on the basis of the proposed PB-ZNN model. The DPB-ZNN algorithm solves a discrete-time DTVCO (DTDTVTO) problem in a fully distributed manner.

The remainder of this study is organized into six sections. In Section 2, some preliminaries concerning graph theory and mathematical notations are provided. Then, in Section 3, the main results of this study are presented. The formulation of the CTDTVCO problem is presented in Section 3.1, and the detailed design process of PB-ZNN model is presented in Section 3.2. The formulation for the DTDTVCO problem and the detailed design process of DPB-ZNN algorithm is presented in Section 3.3. In Section 4, the theoretical proofs of the convergence of both PB-ZNN model and DPB-ZNN algorithm are provided. The efficacy and efficiency of the DPB-ZNN algorithm are illustrated in Section 5 through numerical examples, including a simulation experiment applying it to the cooperative control of redundant manipulators. Finally, the conclusion of this study is given in Section 6.

Before ending this section, the main contributions of this study are listed as follows.

- A novel PB-ZNN model is designed to address the CTDTVCO problem. By incorporating two penalty functions, the PB-ZNN model successfully solves the CTDTVCO problem in a semi-centralized manner.
- On the basis of the PB-ZNN model, a DPB-ZNN algorithm is designed. By incorporating the Euler formula, the proposed DPB-ZNN algorithm effectively solves the DTDTVCO problem in a fully distributed manner.
- The convergence properties of both PB-ZNN model and DPB-ZNN algorithm are proved. Further theoretical analyses prove that the maximum steady-state residual error (MSSRE) for the proposed DPB-ZNN algorithm is of *O*(τ^2^).

## 2 Preliminaries

The notation ||·||_2_ represents the 2-norm of a vector or a matrix. Moreover, (·)^T^ denotes the transpose of a matrix or a vector, *I*_*n*_ is the identity matrix of size *n*×*n*, $1_{d}={\left\{1,\cdots \;,1\right\}}^{\text{T}}\in {\mathbb{R}}^{d}$, and ⊗ represents the Kronecker product (Golub and Loan, 2013).

Some principles from graph theory (Diestel, 2017) are introduced as follows.

The notation G={V,Y,A} denotes a weighted graph, wherein $V=\left\{v_{1},v_{2},\cdots \;,v_{n}\right\}$ represents the set of vertices, and Y⊆V×V denotes the set of edges. The adjacency matrix is denoted by $A={\left(a_{ij}\right)}_{n\times n}\in {\mathbb{R}}^{n\times n}$, where *a*_*ij*_>0 when $\left(v_{i},v_{j}\right)\in Y$ and *a*_*ij*_ = 0 when $\left(v_{i},v_{j}\right)\notin Y$, respectively. Moreover, the notation $N_{i}$ denotes the neighbor set of *i*th vertex. The graph G is said to be connected and undirected when a path between any given pair of vertices within G exists and when $\left(v_{i},v_{j}\right)\in Y$ also indicates $\left(v_{j},v_{i}\right)\in Y$. The degree matrix *D* is defined as $D=\text{diag}\left\{d_{1},d_{2},\cdots \;,d_{n}\right\}\in {\mathbb{R}}^{n\times n}$ where $d_{i}=\overset{n}{\underset{j=1}{\sum }}a_{ij}$. Finally, the Laplacian matrix is defined as *L* = *D*−*A*.

**Assumption 1**: Graph G is undirected and connected.

## 3 Main results

In Subsection 3.1, the formulations of the CTDTVCO problem are provided. To address the CTDTVCO problem, two penalty functions are introduced. In Subsection 3.2, a PB-ZNN model is proposed to solve the CTDTVCO problem in a semi-centralized manner. Then, on the basis of the proposed PB-ZNN model, a novel DPB-ZNN algorithm is proposed to solve the DTDTVCO problem in a fully distributed manner in Subsection 3.3.

### 3.1 CTDTVCO with distributed time-varying equality and inequality constraints

In this study, the CTDTVCO problem within graph G is formulated as


(1) $$\begin{array}{ll} \text{minimize } & f\left(x\left(t\right),t\right)=\overset{n}{\underset{i=1}{\sum }}f_{i}\left(x\left(t\right),t\right), \end{array}$$

(2) $$\begin{array}{ll} \text{subject to } & K_{i}\left(t\right)x\left(t\right)=b_{i}\left(t\right),\;i\in \left\{1,2,\ldots ,n\right\}, \end{array}$$

(3) $$\begin{array}{l} J_{i}\left(t\right)x\left(t\right)\leq c_{i}\left(t\right),\;i\in \left\{1,2,\ldots ,n\right\}, \end{array}$$

where *x*(*t*) ∈ ℝ^*d*^, and $f_{i}\left(x\left(t\right),t\right):{\mathbb{R}}^{d}\times \mathbb{R}\mapsto \mathbb{R}$ denotes the time-varying local objective function of the agent *i* and *f*_*i*_ should be second-order differentiable and strongly convex for all *i* = 1, 2, ⋯ , *n*. Moreover, *K*_*i*_(*t*)*x*(*t*) = *b*_*i*_(*t*) and *J*_*i*_(*t*)*x*(*t*) ≤ *c*_*i*_(*t*) are the time-varying equality and inequality constraints associated with the agent *i*. For all *i* ∈ {1, 2, …, *n*}, the coefficient matrix $K_{i}\left(t\right)\in {\mathbb{R}}^{k_{i}\times d}$ is of full row rank and coefficient matrix *J*_*i*_(*t*) is defined as $J_{i}\left(t\right)=\left[J_{i1}\left(t\right);\ldots ;J_{im_{i}}\left(t\right)\right]\in {\mathbb{R}}^{m_{i}\times d}$. In addition, notations $b_{i}\left(t\right)\in {\mathbb{R}}^{k_{i}}$ and $c_{i}\left(t\right)\in {\mathbb{R}}^{m_{i}}$ denote the coefficient vectors. The above-mentioned matrices and vectors are assumed to be differentiable. It is worth pointing out that all contradictions among agents' equality constraints should be ruled out for the CTDTVCO problem to be solvable.

According to Assumption 1, the communication graph G is both connected and undirected. Hence, the CTDTVCO problem (Equations 1–3) is reformulated as an equivalent problem described by the following Lemma 1.

**Lemma 1**: If graph G is connected and undirected, then the CTDTVCO problem (Equations 1–3) is equivalent to the following optimization problem (Bahman and Jorge, 2014).


(4) $$\begin{array}{ll} \text{minimize } & \varphi \left(\text{x}\left(t\right),t\right)=\overset{n}{\underset{i=1}{\sum }}f_{i}\left(x_{i}\left(t\right),t\right), \end{array}$$

(5) $$\begin{array}{ll} \text{subject to } & \text{J}\left(t\right)\text{x}\left(t\right)\leq \text{c}\left(t\right), \end{array}$$

(6) $$\begin{array}{l} \text{K}\left(t\right)\text{x}\left(t\right)=\text{b}\left(t\right), \end{array}$$

(7) $$\begin{array}{l} \text{L}\text{x}\left(t\right)=0_{nd}, \end{array}$$

where


$$\begin{array}{l} \text{K}\left(t\right)=\left[\begin{array}{cccc} K_{1}\left(t\right) & 0 & \cdots & 0\\ 0 & K_{2}\left(t\right) & \cdots & 0\\ \vdots & \vdots & \ddots & \vdots \\ 0 & 0 & \cdots & K_{n}\left(t\right) \end{array}\right]\in {\mathbb{R}}^{k\times nd},\\ \text{J}\left(t\right)=\left[\begin{array}{cccc} J_{1}\left(t\right) & 0 & \cdots & 0\\ 0 & J_{2}\left(t\right) & \cdots & 0\\ \vdots & \vdots & \ddots & \vdots \\ 0 & 0 & \cdots & J_{n}\left(t\right) \end{array}\right]\in {\mathbb{R}}^{m\times nd}, \end{array}$$

with $\text{x}\left(t\right)=\left[x_{1}\left(t\right);x_{2}\left(t\right);\ldots ;x_{n}\left(t\right)\right]\in {\mathbb{R}}^{nd}$, $k=\overset{n}{\underset{i=1}{\sum }}k_{i}$, and $m=\overset{n}{\underset{i=1}{\sum }}m_{i}$. The coefficient vectors **b**(*t*) and **c**(*t*) are defined as **b**(*t*)= $\left[b_{1}\left(t\right);b_{2}\left(t\right);\ldots ;b_{n}\left(t\right)\right]\in {\mathbb{R}}^{k}$, and $\text{c}\left(t\right)=\left[c_{1}\left(t\right);c_{2}\left(t\right);\ldots ;c_{n}\left(t\right)\right]\in {\mathbb{R}}^{m}$, and the matrix $\text{L}=L⊗I_{d}\in {\mathbb{R}}^{nd\times nd}$. In addition, **0**_*nd*_ denotes an *nd* dimensional vector whose elements are 0.

**Remark 1**: Through Lemma 1, the consensus problem for the CTDTVCO problem (Equations 1–3) is reformulated as an equality constraint (Equation 7). Following the conventional ZNN design method, equality constraints are typically handled by applying the Lagrange function. However, this approach is not suitable for equality constraint (Equation 7), because the Laplacian matrix *L* is inherently rank-deficient. Consequently, setting **L** = *L*⊗*I*_*d*_ also results in a rank-deficient matrix.

To obtain the optimal solution for the CTDTVCO problem (Equations 1–3), two penalty functions are introduced in this study. The definitions of these two penalty functions are as follows:


(8) $$\begin{array}{ll} p_{1}\left(\text{x}\left(t\right)\right) & =\frac{1}{4}\sigma_{1}\overset{n}{\underset{i=1}{\sum }}\underset{j\in N_{i}}{\sum }||x_{i}\left(t\right)-x_{j}\left(t\right)|{|}_{2}^{2}, \end{array}$$

(9) $$\begin{array}{ll} p_{2}\left(\text{x}\left(t\right)\right) & =\rho \overset{n}{\underset{i=1}{\sum }}\overset{m_{i}}{\underset{j=1}{\sum }}{\text{e}}^{-\sigma_{2}N_{j}\left(x_{i}\left(t\right)\right)}, \end{array}$$

where *N*_*j*_(*x*_*i*_(*t*)) = *c*_*ij*_(*t*)−*J*_*ij*_(*t*)*x*_*i*_(*t*) with *c*_*ij*_(*t*) being the *j*th element of vector *c*_*i*_(*t*) and *J*_*ij*_(*t*) being the *j*th row of matrix *J*_*i*_(*t*). In addition, σ_1_, σ_2_>0 are two positive parameters that are sufficiently larege and parameter ρ>0 is positive and near zero.

- The first penalty function intuitively manages information exchange between agents and their neighbors, driving all agents to reach a consensus.
- The second penalty function is from Zhang et al. (2021b), and it addresses the distributed inequality constraints, ensuring that each agent's solution remains bounded.

It is evident that when **Lx**(*t*) → **0**_*nd*_, the value of *p*_1_(**x**(*t*)) approaches zero. Furthermore, when inequality constraints (Equation 5) are met, the value of *p*_2_(**x**(*t*)) becomes a positive number very close to zero. This soft penalty allows slight violations of constraints while still promoting feasible solutions. Conversely, by choosing sufficiently large enough positive parameters σ_1_ and σ_2_, the values of *p*_1_(**x**(*t*)) and *p*_2_(**x**(*t*)) are magnified to serve as punishments for solution **x**(*t*) that fall outside the feasible range. Thus, the CTDTVCO problem (Equations 4–6) is transformed into an equivalent CTDTVCO problem with no inequality constraints as follows:


(10) $$\begin{array}{ll} \text{minimize } & \varphi \left(\text{x}\left(t\right),t\right)+p_{1}\left(\text{x}\left(t\right)\right)+p_{2}\left(\text{x}\left(t\right)\right), \end{array}$$

(11) $$\begin{array}{ll} \text{subject to } & \text{K}\left(t\right)\text{x}\left(t\right)=\text{b}\left(t\right). \end{array}$$

Therefore, by solving the CTDTVCO problem (Equations 10, 11), one acquires an approximate solution to the original CTDTVCO problem (Equations 1–3). To solve the CTDTVCO problem (Equations 10, 11), a Lagrange function is introduced:


(12) $$\begin{array}{l} L\left(\text{x}\left(t\right),\lambda \left(t\right),t\right)=\varphi \left(\text{x}\left(t\right),t\right)+p_{1}\left(\text{x}\left(t\right)\right)+p_{2}\left(\text{x}\left(t\right)\right)\\ \;+\lambda^{\text{T}}\left(t\right)\left(\text{K}\left(t\right)\text{x}\left(t\right)-\text{b}\left(t\right)\right), \end{array}$$

where the Lagrange multiplier λ(*t*) is defined as $\lambda \left(t\right)=\left[\lambda_{1}\left(t\right);\lambda_{2}\left(t\right);\ldots ;\lambda_{n}\left(t\right)\right]\in {\mathbb{R}}^{k}$, with $\lambda_{i}\left(t\right)\in {\mathbb{R}}^{k_{i}}$ being the Lagrange multiplier corresponding to the agent *i*. It is assumed that both ∂*L*(**x**(*t*), λ(*t*), *t*)/∂**x**(*t*) and ∂*L*(**x**(*t*), λ(*t*), *t*)/∂λ(*t*) exist and are continuous. The optimal solution must satisfy the following equations:


(13) $$\{\begin{array}{ll} \frac{\partial L(x(t),\lambda (t),t)}{\partial x(t)}= & \nabla_{x}\varphi (x(t),t)+\sigma_{1}Lx(t)+\Phi (x(t))+K^{\text{T}}(t)\lambda (t)=0_{nd},\\ \frac{\partial L(x(t),\lambda (t),t)}{\partial \lambda (t)}= & K(t)x(t)-b(t)=0_{k}, \end{array}$$

where


$$\begin{array}{l} \nabla_{x}\varphi (x(t),t)=\frac{\partial \varphi (x(t))}{\partial x(t)}=[\nabla_{x}f_{1}(x_{1}(t),t);\nabla_{x}f_{2}(x_{2}(t),t);\ldots ;\\ \;\nabla_{x}f_{n}(x_{n}(t),t)]\in {\mathbb{R}}^{nd}, \end{array}$$

with the elements being


$$\begin{array}{l} \begin{array}{c} \nabla_{x}f_{i}\left(x_{i}\left(t\right),t\right)=\frac{\partial f_{i}\left(x_{i}\left(t\right),t\right)}{\partial x_{i}\left(t\right)}\in {\mathbb{R}}^{d}. \end{array} \end{array}$$

In addition, Φ(**x**(*t*)) is defined as


$$\begin{array}{l} \begin{array}{c} \Phi \left(\text{x}\left(t\right)\right)=\frac{\partial p_{2}\left(\text{x}\left(t\right)\right)}{\partial \text{x}\left(t\right)}=\left[\phi_{1}\left(x_{1}\left(t\right)\right);\phi_{2}\left(x_{2}\left(t\right)\right);\ldots ;\phi_{n}\left(x_{n}\left(t\right)\right)\right]\in {\mathbb{R}}^{nd}, \end{array} \end{array}$$

with


$$\begin{array}{l} \phi_{i}\left(x_{i}\left(t\right)\right)=\rho \sigma_{2}\overset{m_{i}}{\underset{j=1}{\sum }}{\text{e}}^{-\sigma_{2}N_{j}\left(x_{i}\left(t\right)\right)}J_{ij}^{\text{T}}\left(t\right)\in {\mathbb{R}}^{d}. \end{array}$$

For convenience in computation, (Equation 13) is expressed as the following matrix equation:


(14) $$\begin{array}{l} \text{A}\left(t\right)\text{y}\left(t\right)=\text{g}\left(t\right), \end{array}$$

where


$$\begin{array}{l} \text{A}\left(t\right)=\left[\begin{array}{cc} \sigma_{1}\text{L} & {\text{K}}^{\text{T}}\left(t\right)\\ \text{K}\left(t\right) & 0_{k\times k} \end{array}\right]\in {\mathbb{R}}^{\left(nd+k\right)\times \left(nd+k\right)},\\ \text{y}\left(t\right)=\left[\begin{array}{c} \text{x}\left(t\right)\\ \lambda \left(t\right) \end{array}\right]\in {\mathbb{R}}^{\left(nd+k\right)},\\ \text{g}\left(t\right)=\left[\begin{array}{c} -\nabla_{x}\varphi \left(\text{x}\left(t\right),t\right)-\Phi \left(\text{x}\left(t\right)\right)\\ \text{b}\left(t\right) \end{array}\right]\in {\mathbb{R}}^{\left(nd+k\right)}. \end{array}$$

The vector **y**(*t*) needs to be solved at all time. One sees that the CTDTVCO problem (Equations 1–3) is solved if the matrix equation (Equation 14) is solved.

### 3.2 PB-ZNN Model for CTDTVCO Problem Solving

In this subsection, a PB-ZNN model is proposed for the entire system to solve the matrix equation (Equation 14).

To obtain the solution of (Equation 14), an error function is defined as


(15) $$\begin{array}{l} \varepsilon \left(t\right)=\text{A}\left(t\right)\text{y}\left(t\right)-\text{g}\left(t\right)\in {\mathbb{R}}^{nd+k}. \end{array}$$

To minimize the value of the error function ε(*t*) and approach zero, it is essential that the derivative of the error function (Equation 15) with respect to time *t* remains negative (Zhang et al., 2018b). Consequently, the ZNN design formula is employed as follows:


(16) $$\begin{array}{l} \frac{\text{d}\varepsilon \left(t\right)}{\text{d}t}=-\gamma \text{Ψ}\left(\varepsilon \left(t\right)\right), \end{array}$$

where Ψ(·):ℝ^*nd*+*k*^↦ℝ^*nd*+*k*^ is an array consisting of activation functions ψ(·) and a positive parameter γ>0 is used to adjust the convergence rate. It is crucial that the activation function ψ(·) satisfies two properties: It must be monotonically increasing and an odd function. By substituting (Equation 16) into (Equation 15), the following model is obtained:


(17) $$\begin{array}{l} \text{A}\left(t\right)\dot{\text{y}}\left(t\right)=-\dot{\text{A}}\left(t\right)\text{y}\left(t\right)-\gamma \text{Ψ}\left(\text{A}\left(t\right)\text{y}\left(t\right)-\text{g}\left(t\right)\right)-\dot{\text{g}}\left(t\right), \end{array}$$

where $\dot{\text{A}}\left(t\right)$ and $\dot{\text{g}}\left(t\right)$ denote the derivatives with respect to time *t* of the matrix **A**(*t*) and the vector **g**(*t*), respectively.

Moreover, according to Equation 14, one obtains


(18) $$\begin{array}{l} \dot{\text{g}}\left(t\right)=\left[\begin{array}{c} \dot{\delta }\left(t\right)\\ \dot{\text{b}}\left(t\right) \end{array}\right]\in {\mathbb{R}}^{nd+k}, \end{array}$$

where


(19) $$\begin{array}{l} \dot{\delta }\left(t\right)=-\dot{\text{c}}\left(t\right)-\text{H}\left(\text{x}\left(t\right),t\right)\dot{\text{x}}\left(t\right)-\dot{\Phi }\left(\text{x}\left(t\right)\right)\in {\mathbb{R}}^{nd}, \end{array}$$

with $\dot{\text{b}}\left(t\right)=\text{d}\text{b}\left(t\right)/\text{d}t$ and


$$\begin{array}{l} \dot{\Phi }\left(\text{x}\left(t\right)\right)=\left[\frac{\partial^{2}p_{21}\left(x_{1}\left(t\right)\right)}{\partial x_{1}\left(t\right)\partial t};\frac{\partial^{2}p_{22}\left(x_{1}\left(t\right)\right)}{\partial x_{2}\left(t\right)\partial t};\ldots ;\frac{\partial^{2}p_{2n}\left(x_{1}\left(t\right)\right)}{\partial x_{n}\left(t\right)\partial t}\right]\in {\mathbb{R}}^{nd}, \end{array}$$

in which


$$\begin{array}{l} \frac{\partial^{2}p_{2i}(x_{1}(t))}{\partial x_{i}(t)\partial t}=\;\rho \sigma_{2}\sum_{j=1}^{m_{i}}{\text{e}}^{-\sigma_{2}N_{j}(x_{i}(t))}(\sigma_{2}J_{ij}^{\text{T}}(t){\dot{J}}_{ij}(t)x_{i}(t)\\ \;+\;\sigma_{2}J_{ij}^{\text{T}}(t)J_{ij}(t){\dot{x}}_{i}(t)-\sigma_{2}J_{ij}^{\text{T}}(t){\dot{c}}_{ij}+{\dot{J}}_{ij}^{\text{T}}(t)), \end{array}$$

with ${\dot{J}}_{ij}\left(t\right)=\text{d}J_{ij}\left(t\right)/\text{d}t$ and ċ_*ij*_(*t*) = d*c*_*ij*_/d*t*. In addition,


$$\begin{array}{l} \text{H}\left(\text{x}\left(t\right),t\right)=\left[\begin{array}{cccc} H_{1}\left(x_{1}\left(t\right),t\right) & 0 & \cdots & 0\\ 0 & H_{2}\left(x_{2}\left(t\right),t\right) & \cdots & 0\\ \vdots & \vdots & \ddots & \vdots \\ 0 & 0 & \cdots & H_{n}\left(x_{n}\left(t\right),t\right) \end{array}\right]\in {\mathbb{R}}^{nd\times nd}, \end{array}$$

where $H_{i}\left(x_{i}\left(t\right),t\right)\in {\mathbb{R}}^{d\times d}$ for all *i* = 1, 2, …, *n* denotes the Hessian matrix of the agent *i* and the definition of *H*_*i*_(*x*_*i*_(*t*), *t*) is as follows:


$$\begin{array}{l} H_{i}\left(x_{i}\left(t\right),t\right)=\left[\begin{array}{cccc} \frac{\partial^{2}f_{i}\left(x_{i}\left(t\right),t\right)}{\partial x_{i1}^{2}} & \frac{\partial^{2}f_{i}\left(x_{i}\left(t\right),t\right)}{\partial x_{i1}\partial x_{i2}} & \cdots & \frac{\partial^{2}f_{i}\left(x_{i}\left(t\right),t\right)}{\partial x_{i1}\partial x_{id}}\\ \frac{\partial^{2}f_{i}\left(x_{i}\left(t\right),t\right)}{\partial x_{i2}\partial x_{i1}} & \frac{\partial^{2}f_{i}\left(x_{i}\left(t\right),t\right)}{\partial x_{i2}^{2}} & \cdots & \frac{\partial^{2}f_{i}\left(x_{i}\left(t\right),t\right)}{\partial x_{i2}\partial x_{i3}}\\ \vdots & \vdots & \ddots & \vdots \\ \frac{\partial^{2}f_{i}\left(x_{i}\left(t\right),t\right)}{\partial x_{id}\partial x_{i1}} & \frac{\partial^{2}f_{i}\left(x_{i}\left(t\right),t\right)}{\partial x_{id}\partial x_{i2}} & \cdots & \frac{\partial^{2}f_{i}\left(x_{i}\left(t\right),t\right)}{\partial x_{id}^{2}} \end{array}\right]\in {\mathbb{R}}^{d\times d}. \end{array}$$

One notices the simultaneous presence of **x**(*t*) and $\dot{\text{x}}\left(t\right)$ in $\dot{\delta }\left(t\right)$ in Equation 19. This reflects the combined influence of both the state and its time derivative on the dynamics of the system. When numerically computing Equation 17, it becomes necessary to consolidate similar terms. Define two matrices, **M**_1_(*t*) and **M**_2_(*t*), and a vector **h**(*t*), as follows:


$$\begin{array}{l} {\text{M}}_{1}\left(t\right)=\left[\begin{array}{cccc} M_{11}\left(t\right) & 0 & \cdots & 0\\ 0 & M_{12}\left(t\right) & \cdots & 0\\ \vdots & \vdots & \ddots & \vdots \\ 0 & 0 & \cdots & M_{1n}\left(t\right) \end{array}\right]\in {\mathbb{R}}^{nd\times nd},\\ {\text{M}}_{2}\left(t\right)=\left[\begin{array}{cccc} M_{21}\left(t\right) & 0 & \cdots & 0\\ 0 & M_{22}\left(t\right) & \cdots & 0\\ \vdots & \vdots & \ddots & \vdots \\ 0 & 0 & \cdots & M_{2n}\left(t\right) \end{array}\right]\in {\mathbb{R}}^{nd\times nd},\\ \text{h}\left(t\right)=\left[h_{1};h_{2};\ldots ;h_{n}\right]\in {\mathbb{R}}^{nd}, \end{array}$$

where for all *i* = 1, 2, …, *n*,


$$\begin{array}{l} M_{1i}=\rho \sigma_{2}^{2}\overset{m_{i}}{\underset{j=1}{\sum }}{\text{e}}^{-\sigma_{2}N_{j}\left(x_{i}\left(t\right)\right)}J_{ij}^{\text{T}}\left(t\right)J_{ij}\left(t\right)\in {\mathbb{R}}^{d\times d},\\ M_{2i}=\rho \sigma_{2}^{2}\overset{m_{i}}{\underset{j=1}{\sum }}{\text{e}}^{-\sigma_{2}N_{j}\left(x_{i}\left(t\right)\right)}J_{ij}^{\text{T}}\left(t\right){\dot{J}}_{ij}\left(t\right)\in {\mathbb{R}}^{d\times d},\\ h_{i}=\rho \sigma_{2}\overset{m_{i}}{\underset{j=1}{\sum }}{\text{e}}^{-\sigma_{2}N_{j}\left(x_{i}\left(t\right)\right)}\left(-\sigma_{2}J_{ij}^{\text{T}}\left(t\right){ċ}_{ij}+{\dot{J}}_{ij}^{\text{T}}\left(t\right)\right)\in {\mathbb{R}}^{d}, \end{array}$$

Hence, after a restructuring of the ZNN model (Equation 17), one has


$$\begin{array}{l} \text{Q}\left(t\right)\dot{\text{y}}\left(t\right)=-\text{S}\left(t\right)\text{y}\left(t\right)-\gamma \text{Ψ}\left(\text{A}\left(t\right)\text{y}\left(t\right)-\text{g}\left(t\right)\right)+\text{u}\left(t\right), \end{array}$$

where


$$\begin{array}{l} \text{Q}\left(t\right)=\left[\begin{array}{cc} \text{H}\left(\text{x}\left(t\right),t\right)+\sigma_{1}\text{L}+{\text{M}}_{1}\left(t\right) & {\text{K}}^{\text{T}}\left(t\right)\\ \text{K}\left(t\right) & 0_{k\times k} \end{array}\right]\in {\mathbb{R}}^{\left(nd+k\right)\times \left(nd+k\right)},\\ \text{S}\left(t\right)=\left[\begin{array}{cc} {\text{M}}_{2}\left(t\right) & {\dot{\text{K}}}^{\text{T}}\left(t\right)\\ \dot{\text{K}}\left(t\right) & 0_{k\times k} \end{array}\right]\in {\mathbb{R}}^{\left(nd+k\right)\times \left(nd+k\right)},\\ \text{u}\left(t\right)=\left[\begin{array}{c} -\text{h}\left(t\right)-\nabla_{xt}\varphi \left(\text{x}\left(t\right),t\right)\\ \dot{\text{b}}\left(t\right) \end{array}\right]\in {\mathbb{R}}^{nd+k}, \end{array}$$

with $\nabla_{xt}\varphi (x(t),t)=[\partial^{2}f_{1}(x_{1}(t),t)/\partial x_{1}\partial t;\partial^{2}f_{2}(x_{2}(t),t)/\partial x_{2}\partial t;\ldots ;$ $\partial^{2}f_{n}(x_{n}(t),t)/\partial x_{n}\partial t]$. In this study, the linear function (i.e., Ψ(*x*(*t*)) = *x*(*t*)) is chosen as the activation function for simplicity. Therefore, the PB-ZNN model for the whole system is given as


(20) $$\begin{array}{l} \dot{\text{y}}\left(t\right)=-{\text{Q}}^{-1}\left(t\right)\left(\text{S}\left(t\right)\text{y}\left(t\right)-\gamma \left(\text{A}\left(t\right)\text{y}\left(t\right)-\text{g}\left(t\right)\right)+\text{u}\left(t\right)\right). \end{array}$$

Thus, the design of PB-ZNN model that solves the CTDTVCO problem (Equations 1–3) is completed.

It is worth pointing out that, although the PB-ZNN model (Equation 20) is designed to address the CTDTVCO problem, it solves Equation 14 in a semi-decentralized manner. Upon examining matrix **Q**(*t*) in Equation 20, one observes that for agent *i*, due to the presence of **L** in **Q**(*t*), solving the time derivative ẋ_*i*_(*t*) analytically requires information from its neighbors: ẋ_*j*_(*t*) for $j\in N_{i}$. Therefore, if to be analytically solved, the information exchange among agents is distributed, but the PB-ZNN model (Equation 20) is computed in a centralized way.

### 3.3 Distributed PB-ZNN algorithm for DTDTVCO problem solving

In this subsection, a distributed PB-ZNN (DPB-ZNN) algorithm is developed for each agent to solve the DTDTVCO problem in a fully distributed manner.

First, let us consider the following DTDTVCO problem with a computational time interval [*t*_*k*_, *t*_*k*+1_):


(21) $$\begin{array}{ll} \text{minimize } & f\left(x_{k+1},t_{k+1}\right)=\overset{n}{\underset{i=1}{\sum }}f_{i}\left(x_{k+1},t_{k+1}\right), \end{array}$$

(22) $$\begin{array}{ll} \text{subject to } & K_{i_{k+1}}x_{k+1}=b_{i_{k+1}},i\in \left\{1,2,\ldots ,n\right\}, \end{array}$$

(23) $$\begin{array}{l} J_{i_{k+1}}x_{k+1}\leq c_{i_{k+1}},i\in \left\{1,2,\ldots ,n\right\}, \end{array}$$

where *f*(*x*_*k*+1_, *t*_*k*+1_) is generated or measured from the smoothly time-varying signal *f*(*x*(*t*), *t*) by sampling at the time instant *t* = (*k*+1)τ (which is denoted as *t*_*k*+1_), and τ denotes the sampling gap.

The DPB-ZNN algorithm is designed on the basis of the continuous-time PB-ZNN model (Equation 20). Therefore, the distributed form of PB-ZNN model (Equation 20) is given to lay the basis for the DPB-ZNN algorithm.

Through simple matrix computation, the distributed form of equation (Equation 14) for agent *i* to solve in continuous-time is given as


(24) $$\begin{array}{l} A_{i}\left(t\right)y_{i}\left(t\right)=g_{i}\left(t\right), \end{array}$$

where


$$\begin{array}{l} A_{i}\left(t\right)=\left[\begin{array}{cc} \sigma_{1}D_{i} & K_{i}^{\text{T}}\left(t\right)\\ K_{i}\left(t\right) & 0_{k_{i}\times k_{i}} \end{array}\right]\in {\mathbb{R}}^{\left(d+k_{i}\right)\times \left(d+k_{i}\right)},\\ y_{i}\left(t\right)=\left[\begin{array}{c} x_{i}\left(t\right)\\ \lambda_{i}\left(t\right) \end{array}\right]\in {\mathbb{R}}^{d+k_{i}},\\ g_{i}\left(t\right)=\left[\begin{array}{c} -\nabla_{x}f_{i}\left(x_{i}\left(t\right),t\right)-\phi \left(x_{i}\left(t\right)\right)+\sigma_{1}e_{i}\left(t\right)\\ b_{i}\left(t\right) \end{array}\right]\in {\mathbb{R}}^{d+k_{i}}, \end{array}$$

with *D*_*i*_ = *d*_*i*_*I*_*d*×*d*_ and *d*_*i*_ denotes the degree of the Laplacian matrix *L* associated with the agent *i*. In addition, $e_{i}\left(t\right)=\underset{j\in N_{i}}{\sum }a_{ij}x_{j}\left(t\right)$. It is evident that the distributed solution [ẋ_1_(*t*);ẋ_2_(*t*);…;ẋ_*n*_(*t*)] for equation (Equation 24) is equivalent to $\dot{\text{x}}\left(t\right)$ in equation (Equation 14).

Consequently, for the agent *i*, the distributed form of the error function is defined as


(25) $$\begin{array}{l} \varepsilon_{i}\left(t\right)=A_{i}\left(t\right)y_{i}\left(t\right)-g_{i}\left(t\right)\in {\mathbb{R}}^{d+k_{i}}. \end{array}$$

Therefore, following the same design process as the PB-ZNN model, one has


(26) $$\begin{array}{l} Q_{i}\left(t\right)\dot{y_{i}}\left(t\right)=-S_{i}\left(t\right)y_{i}\left(t\right)-\gamma \text{Ψ}\left(A_{i}\left(t\right)y_{i}\left(t\right)-g_{i}\left(t\right)\right)+u_{i}\left(t\right), \end{array}$$

where


$$\begin{array}{l} Q_{i}\left(t\right)=\left[\begin{array}{cc} H_{i}\left(x_{i}\left(t\right)\right)+\sigma_{1}D_{i}+M_{1i}\left(t\right) & K_{i}^{\text{T}}\left(t\right)\\ K_{i}\left(t\right) & 0_{k_{i}\times k_{i}} \end{array}\right]\in {\mathbb{R}}^{\left(d+k_{i}\right)\times \left(d+k_{i}\right)},\\ S_{i}\left(t\right)=\left[\begin{array}{cc} M_{2i}\left(t\right) & {\dot{K_{i}}}^{\text{T}}\left(t\right)\\ \dot{K_{i}}\left(t\right) & 0_{k_{i}\times k_{i}} \end{array}\right]\in {\mathbb{R}}^{\left(d+k_{i}\right)\times \left(d+k_{i}\right)},\\ u_{i}\left(t\right)=\left[\begin{array}{c} -h_{i}\left(t\right)-\dot{\nabla_{x}}f_{i}\left(x_{i}\left(t\right),t\right)+\sigma_{1}{ė}_{i}\left(t\right)\\ \dot{b_{i}}\left(t\right) \end{array}\right]\in {\mathbb{R}}^{d+k_{i}}, \end{array}$$

with ${ė}_{i}\left(t\right)=\underset{j\in N_{i}}{\sum }a_{ij}{ẋ}_{j}\left(t\right)$. In addition, *M*_1*i*_(*t*), *M*_2*i*_(*t*), and *h*_*i*_(*t*) are defined in the same way as in Section 3.2. Therefore, for agent *i*, the distributed form of PB-ZNN model (Equation 20) is formulated as


$$\begin{array}{l} \dot{y_{i}}\left(t\right)=-\gamma Q_{i}^{-1}\left(t\right)\text{Ψ}\left(A_{i}\left(t\right)y_{i}\left(t\right)-g_{i}\left(t\right)\right)-Q_{i}^{-1}\left(t\right)S_{i}\left(t\right)y_{i}\left(t\right)\\ \;+Q_{i}^{-1}\left(t\right)u_{i}\left(t\right). \end{array}$$

Hence, the distributed discrete-time PB-ZNN model is formulated as


(27) $${\dot{y}}_{i_{k}}=-\gamma Q_{i_{k}}^{-1}\Psi \left(A_{i_{k}}y_{i_{k}}-g_{i_{k}}\right)-Q_{i_{k}}^{-1}S_{i_{k}}y_{i}+Q_{i_{k}}^{-1}u_{i_{k}},$$

where *Q*_*i*_*k*__, *A*_*i*_*k*__, *S*_*i*_*k*__, *g*_*i*_*k*__, and *u*_*i*_*k*__ are generated or measured from the smoothly time-varying signals *Q*_*i*_(*t*), *A*_*i*_(*t*), *S*_*i*_(*t*), *g*_*i*_(*t*), and *u*_*i*_(*t*), respectively. Upon examining *u*_*i*_*k*__, one notices that ${ė}_{i_{k}}=\underset{j\in N_{i}}{\sum }a_{ij}{ẋ}_{j_{k}}$ is required for agent *i* to compute ẋ_*i*_*k*__. However, ẋ_*j*_*k*__ is unknown to agent *i* in a fully distributed manner. Therefore, an approximation for ė_*i*_*k*__ is introduced.

In traditional methods that solve distributed optimization problems, agents exchange *x*_*i*_*k*__ with their neighbors to address the consensus problem. In this study, an alternative approach is proposed. Instead of merely exchanging the information *x*_*i*_*k*__, each agent maintains a short memory of their neighbors' states. By using the Euler formula, agents approximate ẋ_*j*_*k*__ for $j\in N_{i}$ on the basis of the memories that they kept, and ultimately approximate ė_*i*_*k*__ effectively.

The Euler formula used in this study is given as follows (Chen et al., 2024).


(28) $$\begin{array}{l} {\dot{x}}_{i_{k}}={\dot{\tilde{x}}}_{i_{k}}+O\left(\tau \right)=\frac{x_{i_{k}}-x_{i_{k-1}}}{\tau }+O\left(\tau \right). \end{array}$$

This formula approximates the derivative ẋ_*i*_*k*__ using the backward difference of *x*_*i*_*k*__ over the time step τ. The term *O*(τ) represents the truncation error, indicating that the approximation becomes more accurate as τ approaches zero. Therefore, the approximation of ė_*i*_(*t*) is defined as


(29) $${\dot{e}}_{i_{k}}={\dot{\tilde{e}}}_{i_{k}}+O(\tau )=\sum_{j\in N_{i}}{\dot{\tilde{x}}}_{j_{k}}+O(\tau ).$$

Here, ė_*i*_*k*__ is approximated by summing the estimated derivatives of the neighbors' states ${\dot{\tilde{x}}}_{j_{k}}$, utilizing the stored state information to eliminate the need for continuous communication. Hence, the fully distributed DPB-ZNN algorithm that solves the DTDTVCO problem is given as


$$\begin{array}{l} {\dot{y}}_{i_{k}}≐-\gamma Q_{i_{k}}^{-1}\text{Ψ}\left(A_{i_{k}}y_{i_{k}}-g_{i_{k}}\right)-Q_{i_{k}}^{-1}S_{i_{k}}y_{i}+Q_{i_{k}}^{-1}{\tilde{u}}_{i_{k}}, \end{array}$$

with


$${\tilde{u}}_{i_{k}}=\left[\begin{array}{c} -h_{i_{k}}-\dot{\nabla_{x}}f_{i}(x_{i_{k}},t_{k})+\sigma_{1}{\dot{\tilde{e}}}_{i_{k}}\\ {\dot{b}}_{i_{k}} \end{array}\right].$$

Hence, by choosing the linear function as the active function, one obtains


$$\begin{array}{l} {\dot{y}}_{i_{k}}≐-\gamma Q_{i_{k}}^{-1}\left(A_{i_{k}}y_{i_{k}}-g_{i_{k}}\right)-Q_{i_{k}}^{-1}S_{i_{k}}y_{i_{k}}+Q_{i_{k}}^{-1}{\tilde{u}}_{i_{k}}. \end{array}$$

By introducing the 2-step time-discretization (TD) formula (Chen et al., 2024).


(30) $$\begin{array}{l} {\dot{\xi }}_{k}=\frac{1}{\tau }\left(\xi_{k+1}-\xi_{k}\right)+O\left(\tau \right), \end{array}$$

the DPB-ZNN algorithm is obtained as


(31) $$\begin{array}{ll} y_{i_{k+1}}≐ & y_{i_{k}}-hQ_{i_{k}}^{-1}\left(A_{i_{k}}y_{i_{k}}-g_{i_{k}}\right)-\tau Q_{i_{k}}^{-1}S_{i_{k}}y_{i_{k}}+\tau Q_{i_{k}}^{-1}{\tilde{u}}_{i_{k}}, \end{array}$$

where parameter *h* = τγ is used to scale the convergence rate. The proposed DPB-ZNN algorithm (Equation 31) solves the DTDTVCO problem in a fully distributed manner. Notably, information exchange between agents is strictly limited to their solutions *x*_*i*_*k*__. Local information, including each agent's own objective function and constraints, remains inaccessible to its neighbors.

## 4 Theoretical analyses

In this section, the convergence theorems for the PB-ZNN model (Equation 31) and DPB-ZNN algorithm (Equation 26) are established and proved.

### 4.1 Convergence Theorem of PB-ZNN Model

On the basis of the analyses presented in Subsection 3.1, the CTDTVCO problem (Equations 1–3) is reformulated as the matrix equation (Equation 15). Therefore, solving the CTDTVCO (Equations 1–3) is equivalent to solving the matrix equation (Equation 15).

**Theorem 1**: For the CTDTVCO problem (Equations 1–3), consider that a monotonically increasing odd activation function Ψ(·) is used. Starting from any initial state **y**(0) ∈ ℝ^*nd*+*k*^, the residual error ||ε(*t*)||_2_ converges to zero, meaning $\lim_{t\to +\infty }||\varepsilon \left(t\right)|{|}_{2}=0$.

**Proof**: Define a Lyapunov candidate function as


(32) $$\begin{array}{l} V\left(t\right)=\frac{||\varepsilon \left(t\right)|{|}_{2}^{2}}{2}=\varepsilon^{\text{T}}\left(t\right)\varepsilon \left(t\right)\geq 0. \end{array}$$

Hence, the time derivative of *V*(*t*) is


(33) $$\begin{array}{l} \dot{V}\left(t\right)=-\gamma \overset{nd+k}{\underset{i=1}{\sum }}\varepsilon_{i}\left(t\right)\psi \left(\varepsilon_{i}\left(t\right)\right). \end{array}$$

With Ψ(·) being monotonically increasing and odd, the following conditions hold true for ψ_*i*_(·):


(34) $$\psi (\varepsilon_{i}(t))\{\begin{array}{l} >0,\text{ if }\varepsilon_{i}(t)>0\\ =0,\text{ if }\varepsilon_{i}(t)=0\\ <0,\text{ if }\varepsilon_{i}(t)<0 \end{array}.$$

Therefore, it is guaranteed that ε_*i*_(*t*)ψ(ε_*i*_(*t*)) ≥ 0 always holds true, which means $\dot{V}\left(t\right)\leq 0$. According to the Lyapunov stability theorem (Khalil, 2002), one obtains that ||ε(*t*)||_2_ converges to zero, meaning $\lim_{t\to +\infty }||\varepsilon \left(t\right)|{|}_{2}=0$. Thus, the proof is completed.  ■

### 4.2 Convergence theorem of DPB-ZNN algorithm

In this subsection, detailed theoretical analyses about the DPB-ZNN algorithm (Equation 31) are given. The DPB-ZNN algorithm is deemed as a linear 2-step method. For better understanding, some lemmas are introduced as follows (Chen et al., 2024).

**Lemma 2**: A linear *N*-step method is formulated as $\overset{N}{\underset{j=0}{\sum }}\omega_{j}\alpha_{k+j}=\tau \overset{N}{\underset{j=0}{\sum }}\upsilon_{j}\beta_{k+j}$. The first and second characteristic polynomials of the linear multiple-step method are $z\left(\iota \right)=\overset{N}{\underset{j=0}{\sum }}\omega_{j}\iota^{j}$ and $\zeta \left(\iota \right)=\overset{N}{\underset{j=0}{\sum }}\upsilon_{j}\iota^{j}$, respectively. If all complex roots of the characteristic polynomial *z*(ι) ensure |ι| ≤ 1, and if there exists |ι| = 1 with the root that ensures |ι| = 1 is simple, then the corresponding *N*-step method is 0-stable.

**Lemma 3**: A linear *N*-step method is formulated as $\overset{N}{\underset{j=0}{\sum }}\omega_{j}\alpha_{k+j}=\tau \overset{N}{\underset{j=0}{\sum }}\upsilon_{j}\beta_{k+j}$. The order of truncation error when synthesizing the *N*-step method can be checked by computing $w_{0}=\overset{N}{\underset{j=0}{\sum }}\omega_{j}$ and $w_{j}=\overset{N}{\underset{j=1}{\sum }}j^{i}/i!\omega_{j}-\overset{N}{\underset{j=0}{\sum }}j^{i-1}/\left(i-1\right)!\upsilon_{j}$. If *w*_*q*_ ≠ 0 and *w*_*i*_ = 0 with *i* < *q*, the *N*-step method, then the multiple-step method has truncation error of *O*(τ^*q*^).

**Lemma 4**: A linear *N*-step method is formulated as $\overset{N}{\underset{j=0}{\sum }}\omega_{j}\alpha_{k+j}=\tau \overset{N}{\underset{j=0}{\sum }}\upsilon_{j}\beta_{k+j}$. The first and second characteristic polynomials of the linear multiple-step method are $z\left(\iota \right)=\overset{N}{\underset{j=0}{\sum }}\omega_{j}\iota^{j}$ and $\zeta \left(\iota \right)=\overset{N}{\underset{j=0}{\sum }}\upsilon_{j}\iota^{j}$, respectively. If *z*(1) = 0 and ż(1) = ζ(1), with ż being the derivative of *z*(ι), the *N*-step method is consistent.

**Lemma 5**: A linear *N*-step method is formulated as $\overset{N}{\underset{j=0}{\sum }}\omega_{j}\alpha_{k+j}=\tau \overset{N}{\underset{j=0}{\sum }}\upsilon_{j}\beta_{k+j}$. The *N*-step method is convergent, if and only if Lemmas 1 and 3 are satisfied. That is, an *N*-step method is convergent, if and only if it is 0-stable and consistent.

On the basis of the above lemmas, the theorem about the convergence property of DPB-ZNN algorithm (Equations 24) is proved.

**Theorem 2**: Consider the CTDTVO problem (Equations 1–3). Suppose that for every agent *i*, its local objective function *f*_*i*_(*x*_*i*_(*t*), *t*) has continuous 2nd order derivatives. With design parameter γ > 0 and sufficiently small sampling interval gap, the DPB-ZNN algorithm (Equation 31) is convergent with a truncation error *O*(τ^2^).

**Proof**: According to Lemma 5, if and only if the DPB-ZNN algorithm (Equation 24) is 0-stable and consistent, the DPB-ZNN algorithm (Equation 24) is convergent.

According to Lemma 2, the first characteristic polynomial of the DPB-ZNN algorithm (Equation 24) is formulated as


(35) $$\begin{array}{l} z_{2}\left(\iota \right)=\iota -1. \end{array}$$

The root of the characteristic polynomial is ι = 1. One obtains that all roots ensure |ι| ≤ 1 and when|ι| = 1 the root is simple. Therefore, the DPB-ZNN algorithm (Equation 24) is 0-stable.

On the basis of (Equations 30, 31), the following equation for the DPB-ZNN algorithm (Equation 31) is formulated:


(36) $$\begin{array}{l} y_{i_{k+1}}=y_{i_{k}}+\tau \left({\dot{y}}_{i_{k}}+\sigma_{1}O\left(\tau \right)\right)+\tau O\left(\tau \right)\\ \;=y_{i_{k}}+\tau {\dot{y}}_{i_{k}}+O\left(\tau^{2}\right). \end{array}$$

According to Lemma 3, $w_{j}=\overset{N}{\underset{j=1}{\sum }}j^{i}/i!\omega_{j}-\overset{N}{\underset{j=0}{\sum }}j^{i-1}/\left(i-1\right)!\upsilon_{j}$. Hence, *w*_2_ is obtained as


$$\begin{array}{l} w_{2}=\frac{2^{2}}{2!}-\frac{1^{2}}{2!}-\frac{1^{1}}{1!}=\frac{1}{2}\neq 0. \end{array}$$

One computes that *w*_*j*_ = 0 for *j* = 0, 1. Therefore, according to Lemma 3, the DPB-ZNN algorithm (Equation 31) has a truncation error order *O*(τ^2^). In addition, the second characteristic polynomial of the DPB-ZNN algorithm (Equation 31) is formulated as ζ(ι) = 1. Hence, one has ż(1) = ζ(1) = 1. Thus, according to Lemma 4, the DPB-ZNN algorithm (Equation 31) is consistent order *O*(τ^2^).

According to Lemma 5, the DPB-ZNN algorithm (Equation 31) is convergent since it is 0-stable and consistent order *O*(τ^2^). Thus, the proof is completed.     ■

**Theorem 3**: Consider the CTDTVO problem (Equations 1–3). Suppose that, for every agent *i*, its local objective function *f*_*i*_(*x*_*i*_(*t*), *t*) have continuous 2nd order derivatives. With design parameter γ>0 and sufficiently small sampling interval gap, the maximum steady-state residual error (MSSRE) synthesized by the DPB-ZNN algorithm (Equation 26) $\lim_{k\to +\infty }\text{sup}||\varepsilon_{i_{k}}|{|}_{2}$ is of *O*(τ^2^).

**Proof**: Let $y_{i_{k+1}}^{*}$ denotes the actual solution to the problem, i.e., ε_*i*_*k*+1__ = 0 when $y_{i_{k+1}}=y_{i_{k+1}}^{*}$. According to Theorem 3, the DPB-ZNN algorithm (Equation 31) has a truncation error of *O*(τ^2^), meaning $y_{i_{k+1}}=y_{i_{k+1}}^{*}+O\left(\tau^{2}\right)$. By applying the Taylor expansion, one further has


$$\begin{array}{l} \varepsilon_{i_{k+1}}\left(y_{i_{k+1}},t_{k+1}\right)=\varepsilon_{i_{k+1}}\left(y_{i_{k+1}}^{*}+O\left(\tau^{2}\right),t_{k+1}\right)\\ \;=\varepsilon_{i_{k+1}}\left(y_{i_{k+1}}^{*},t_{k+1}\right)+\frac{\partial \varepsilon_{i_{k+1}}\left(y_{i_{k+1}},t_{k+1}\right)}{\partial y_{i_{k+1}}}O\left(\tau^{2}\right)\\ \;+O\left(\tau^{4}\right)\\ \;\leq \frac{\partial \varepsilon_{i_{k+1}}\left(y_{i_{k+1}},t_{k+1}\right)}{\partial y_{i_{k+1}}}O\left(\tau^{2}\right). \end{array}$$

Hence, the MSSRE is deducted as


$$\begin{array}{r} \underset{k\to +\infty }{\text{lim}}\text{sup}{\left\|\varepsilon_{i_{k+1}}(y_{i_{k+1}},t_{k+1})\right\|}_{2}\leq \underset{k\to +\infty }{\text{lim}}\text{sup}{\left\|\frac{\partial \varepsilon_{i_{k+1}}(y_{i_{k+1}}^{*},t_{k+1})}{\partial y_{i_{k+1}}^{*}}O(\tau^{2})\right\|}_{2}\\ \leq \underset{k\to +\infty }{\text{lim}}\text{sup}{\left\|\frac{\partial \varepsilon_{i_{k+1}}(y_{i_{k+1}}^{*},t_{k+1})}{\partial y_{i_{k+1}}^{*}}\right\|}_{\text{F}}O(\tau^{2}), \end{array}$$

where||·||_F_ represents the Frobenius norm of a matrix. Since $y_{i_{k+1}}^{*}$ is the actual solution, the partial derivative $||\partial \varepsilon_{k+1}^{i}\left(y_{i_{k+1}}^{*},t_{k+1}\right)/\partial y_{i_{k+1}}^{*}|{|}_{\text{F}}$ evaluates to a constant matrix, bounded due to the continuity of the second-order derivatives of *f*_*i*_. Therefore, the MSSRE synthesized by the DPB-ZNN algorithm (Equation 31) is of *O*(τ^2^). Thus, the proof is completed.   ■

## 5 Illustrative examples

In this section, two numerical examples are presented. These examples serve to validate the effectiveness of the proposed DPB-ZNN algorithm (Equation 31) discussed in this study.

### 5.1 Example 1: DTDTVCO problem by DPB-ZNN algorithm

**Figure 1:**
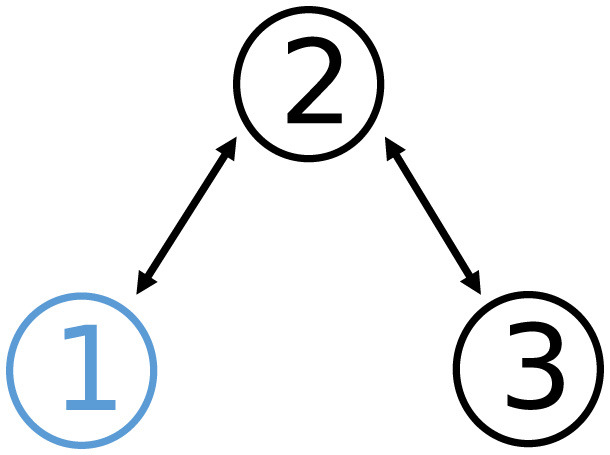
Communication topology of graph $G_{1}$ in Example 1, where $G_{1}$ is connected and undirected.

Consider a DTDTVCO problem with a MAS consisting of three agents. The formulation of the DTDTVCO problem is given as follows:


(37) $$\begin{array}{l} \text{minimize }f\left({\text{x}}_{k+1},t_{k+1}\right)=\overset{3}{\underset{i=1}{\sum }}f_{i}\left(x_{i_{k+1}},t_{k+1}\right),\\ \text{subject to }K_{i_{k+1}}{\text{x}}_{k+1}=b_{i_{k+1}},\;i=1,\\ \;J_{i_{k+1}}{\text{x}}_{k+1}\leq c_{i_{k+1}},\;i=2,3, \end{array}$$

where $x_{i_{k}}\in {\mathbb{R}}^{2}$. The communication topology of the network $G_{1}$ is shown in Figure 1. The detailed time-varying local objective functions *f*_*i*_ and the corresponding time-varying local constraints are given through Tables 1, 2.

**Table 1:** Expressions of time-varying objective functions of all agents in Example 1.

**Objective function**	**Expression**
*f*_1_(*x*_*k*_, *t*_*k*_)	$\frac{\sin\left(t_{k}\right)}{4}x_{1_{k}}^{2}+x_{2_{k}}^{2}+\cos\left(t_{k}\right)x_{1_{k}}x_{2_{k}}$
*f*_2_(*x*_*k*_, *t*_*k*_)	$x_{1_{k}}^{2}+\frac{\sin\left(t_{k}\right)}{4}x_{2_{k}}^{2}+\cos\left(3t_{k}\right)x_{1_{k}}$
*f*_3_(*x*_*k*_, *t*_*k*_)	$x_{1_{k}}^{2}+x_{2_{k}}^{2}+\sin\left(3t_{k}\right)x_{2_{k}}$

**Table 2:** Expressions of time-varying constraints of all agents in Example 1.

**Constraint**	**Expressions**
*K*_1_*k*__*x*_1_*k*__ = *b*_1_*k*__	sin(*t*_*k*_)*x*_1_*k*__+cos(*t*_*k*_)*x*_2_*k*__ = cos(2*t*_*k*_)
*J*_2_*k*__*x*_2_*k*__ ≤ *c*_2_*k*__	*x*_1_*k*__ ≤ 0.5cos(*t*_*k*_−4.4)+1.5 *x*_2_*k*__ ≤ 0.5cos(*t*_*k*_−4)+0.9
*J*_3_*k*__*x*_3_*k*__ ≤ *c*_3_*k*__	*x*_1_*k*__ ≥ −0.5cos(*t*_*k*_−3.6)−1 *x*_2_*k*__ ≥ −0.5cos(*t*_*k*_−3.6)−1

Before conducting the experiment, the parameters of the DPB-ZNN algorithm (Equation 31) must be properly configured. The total solving time is set to *T* = 10 s, the sampling gap τ is set to τ = 0.001 s, and *h* is set to 0.2. In this experiment, for the first penalty function, σ_1_ is set to 50. To guarantee that *x*_*i*_*k*__ remains within the feasible region of the inequality constraints for any agent, σ_2_ is set to 100, while the parameter ρ is set to a value close to zero, specifically 0.001.

**Figure 2:**
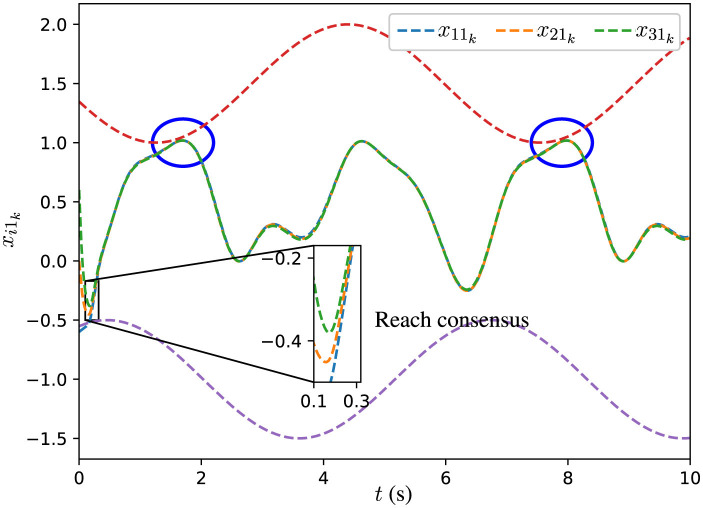
Trajectories of *x*_*i*_1__*k*__ for all agents synthesized by DPB-ZNN algorithm (Equation 31).

**Figure 3:**
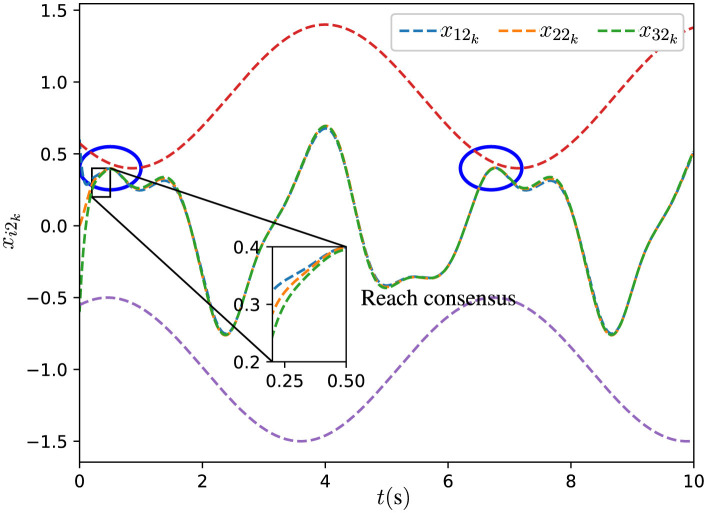
Trajectories of *x*_*i*_2__*k*__ for all agents synthesized by DPB-ZNN algorithm (Equation 31).

The initial states *x*_*i*_0__ are set as $x_{1_{0}}={\left[-0.6,0.6\right]}^{\text{T}}$, $x_{2_{0}}={\left[0,0\right]}^{\text{T}}$, and $x_{3_{0}}={\left[0.6,-0.6\right]}^{\text{T}}$. The experimental results are illustrated through Figures 2, 3. In Figures 2, 3, the dashed red lines denote the inequality constraints *J*_2_*k*__*x*_2_*k*__ ≤ *c*_2_*k*__ associated with the agent 2, and dashed purple lines denote the inequality constraints *J*_3_*k*__*x*_3_*k*__ ≤ *c*_3_*k*__ associated with the agent 3.

From Figures 2, 3, one sees that the three agents achieve consensus from different initial states. In Figures 2, 3, the distributed time-varying inequality constraints are denoted by the red and purple dashed lines. As pointed out by the blue circles in Figures 2, 3, for each agent, their solutions are constrained by the distributed time-varying inequality constraints.

**Figure 4:**
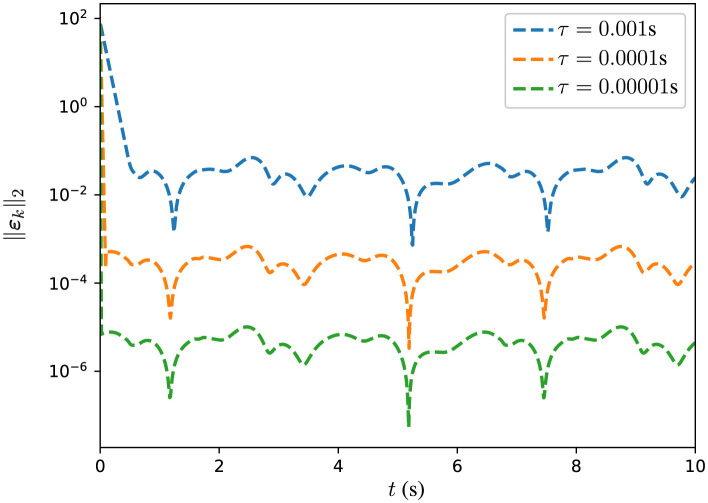
Trajectories of MSSRE ||ε_*k*_||_2_ for DPB-ZNN algorithm in solving DTDTVCO problem (Equation 40), with different τ.

In addition, several experiments are conducted with DPB-ZNN algorithm (Equation 31) solving the DTDTVCO problem (Equation 37) with different τ. The corresponding MSSREs ||ε_*k*_||_2_ are presented in Figure 4, Table 3. In Figure 4, the MSSREs ||ε_*k*_||_2_ with different τ are presented. Figure 4 includes three distinct trajectories corresponding to τ = 0.001 s (blue dashed line), τ = 0.0001 s (orange dashed line), and τ = 0.00001 s (green dashed line). The blue dashed line, for τ = 0.001 s, shows a decreasing MSSRE starting from approximately 10^2^ to approximately 10^−2^. The orange dashed line, for τ = 0.0001 s, reflects a faster convergence with MSSRE reducing to 10^−4^. The green dashed line, for τ = 0.00001 s, presents the most rapid reduction, with MSSRE decreasing from 10^2^ to 10^−6^, highlighting the advantage of very small time steps for precise updates. The figure reveals a clear trend: As τ decreases, the MSSRE also decreases, indicating improved performance of the algorithm with smaller τ values. From Figure 4, Table 3, one sees that the MSSRE ||ε_*k*_||_2_ is of *O*(τ^2^), which corroborates the theoretical analyses.

**Table 3:** MSSREs of DPB-ZNN algorithm (Equation 31) in solving DTDTVCO problem (Equation 37) with different τ.

**τ**	**MSSRE ||ε_*k*_||_2_**
0.001 s	3.76 × 10^−2^
0.0001 s	5.51 × 10^−4^
0.00001 s	6.44 × 10^−6^

Moreover, the gradient neural network (GNN) algorithm is often used to solve time-varying problems with constraints. Therefore, a comparison experiment between the GNN and the proposed DPB-ZNN algorithm is conducted.

First, for the agent *i*, a scalar-valued energy function is designed as follows:


(38) $$\begin{array}{l} e_{i_{k}}^{G}=\frac{1}{2}||\varepsilon_{i_{k}}|{|}_{2}^{2}, \end{array}$$

where ε_*i*_*k*__ = *A*_*i*_*k*__*y*_*i*_*k*__−*g*_*i*_*k*__. Then, by exploiting the gradient information of the energy function (Equation 38), one obtains


$$\begin{array}{l} {\dot{y}}_{i_{k}}=-\gamma^{G}\frac{\partial e_{i_{k}}^{G}}{\partial y_{i_{k}}}=-\gamma^{G}{\left(\frac{\partial \varepsilon_{i_{k}}}{\partial y_{i_{k}}}\right)}^{\text{T}}\varepsilon_{i_{k}}=-\gamma^{G}A_{i_{k}}^{\text{T}}\left(A_{i_{k}}y_{i_{k}}-g_{i_{k}}\right), \end{array}$$

where the parameter γ^*G*^ is used to scale the convergence rate of the GNN algorithm (Zhang et al., 2021b). Hence, the GNN algorithm for solving the DTDTVCO problem is given as


(39) $$\begin{array}{l} y_{i_{k+1}}=y_{i_{k}}-\gamma^{G}\tau A_{i_{k}}^{\text{T}}\left(A_{i_{k}}y_{i_{k}}-g_{i_{k}}\right), \end{array}$$

**Figure 5:**
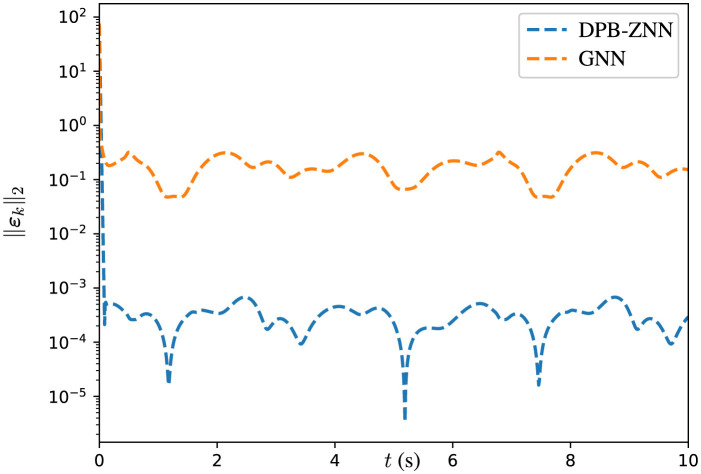
Trajectories of ||ε_*k*_||_2_ of GNN and DPB-ZNN algorithms in solving DTDTVCO problem.

Then, both the GNN algorithm (Equation 39) and the DPB-ZNN algorithm (Equation 31) are used to solve the DTDTVCO problem (Equation 37). For both the GNN algorithm and the DPB-ZNN algorithm, the parameters σ_1_, σ_2_, ρ, and the initial states are set the same. The corresponding experimental results are shown in Table 4, Figure 5.

**Table 4:** Comparisons between GNN algorithm and DPB-ZNN algorithm in solving DTDTVCO problem.

**Algorithm**	**Parameter**	**τ**	**MSSRE ε_*k*_||_2_**
GNN	γ^*G*^ = 2, 000	0.0001s	2.16 × 10^−1^
DPB-ZNN	γ = 2, 000	0.0001s	3.54 × 10^−4^

The MSSRE ||ε_*k*_||_2_ along with the corresponding parameters for both GNN and DPB-ZNN algorithms are provided in Table 4. From Table 4 and Figure 5, one sees that, with the sampling time being the same τ = 0.0001 s, DPB-ZNN algorithm (Equation 31) achieves a higher accuracy with the MSSRE ||ε_*k*_||_2_ being 3.54 × 10^−4^, while the MSSRE ||ε_*k*_||_2_ of GNN algorithm is 2.16 × 10^−1^. To sum up, the DPB-ZNN algorithm (Equation 31) has a higher accuracy when solving DTDTVCO problem than the GNN algorithm.

**Figure 6:**
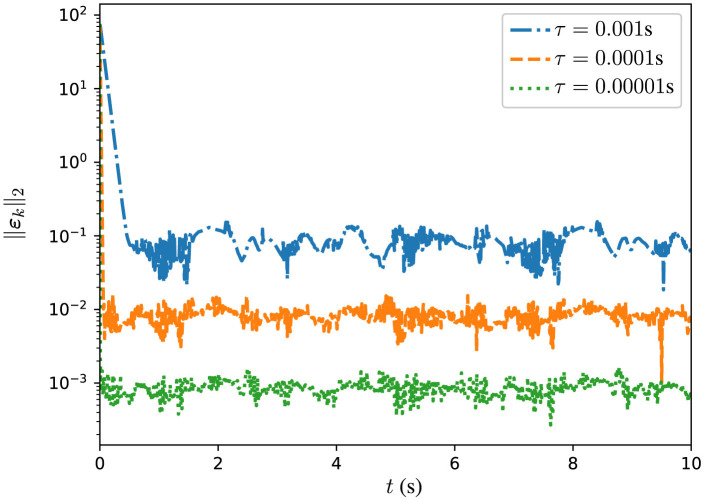
Trajectories of MSSRE ||ε_*k*_||_2_ for DPB-ZNN algorithm in solving DTDTVCO problem with random noise and different τ.

In addition, to investigate the impact of model inaccuracies and measurement noise, numerical experiments on a DTVCO problem with noise, solved by the DPB-ZNN algorithm, are conducted. To lay the basis for further investigation on the robustness of DPB-ZNN algorithm under the pollution of unknown noises, one obtains the following equation:


(40) $$\begin{array}{ll} y_{i_{k+1}}≐ & y_{i_{k}}-hQ_{i_{k}}^{-1}\left(A_{i_{k}}y_{i_{k}}-g_{i_{k}}\right)-\tau Q_{i_{k}}^{-1}S_{i_{k}}y_{i_{k}}+\tau Q_{i_{k}}^{-1}{\tilde{u}}_{i_{k}}+\varrho_{i_{k}}, \end{array}$$

where ϱ_*i*_*k*__ ∈ [−0.5, 0.5] denotes a discrete-time bounded unknown random noise that is uniformly distributed within the range [−0.5, 0.5]. The corresponding experimental results are presented in Figure 6. Except for the bounded random noise ϱ_*i*_*k*__, the initial states and corresponding parameters are set to be the same as those in Subsection 5.1. The trajectories of the residual error ||ε_*k*_||_2_ of the DPB-ZNN algorithm with different values of τ are illustrated in Figure 6.

From the Figure 6, one observes that the MSSRE converges over time for all three sampling intervals, indicating that the DPB-ZNN algorithm effectively reduces the residual errors in the presence of noise. The trajectory with τ = 0.00001 s (green dash line) shows the fastest convergence, reaching the lowest error value within the shortest time, with MSSRE values dropping to approximately 10^−4^. The trajectory with τ = 0.0001 s (orange dashed line) also converges well but at a slightly slower rate, with MSSRE values reaching approximately 10^−3^. The trajectory with τ = 0.001 s (blue dashed line) converges the slowest, taking the longest time to reach a low error value, with MSSRE values approximately 10^−2^.

These experimental results illustrate the robustness of the DPB-ZNN algorithm as it consistently minimizes residual errors and converges effectively even in the presence of noise. The smaller sampling intervals τ lead to faster convergence and lower residual errors, highlighting the algorithm's ability to handle noise polluted time-varying challenges with high accuracy and efficiency.

### 5.2 Example 2: DPB-ZNN algorithm application to cooperative control of redundant robot manipulators

**Figure 7:**
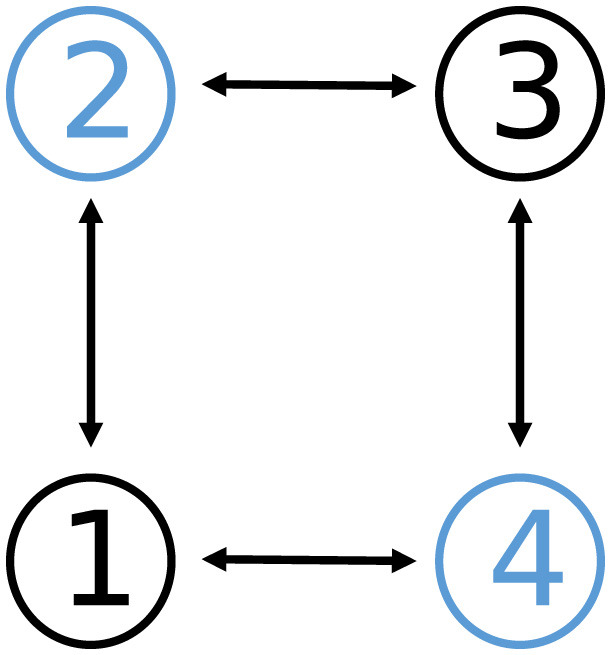
Communication topology of graph $G_{2}$ in Example 3, where $G_{2}$ is connected and undirected.

To illustrate the efficacy of the proposed DPB-ZNN algorithm (Equation 31) in applications, a cooperative control experiment is simulated. In this experiment, we consider a group of four identical 5-joint planar redundant robot manipulators performing a cooperative control task. The communication topology of the four robot manipulators is shown in Figure 7.

**Figure 8:**
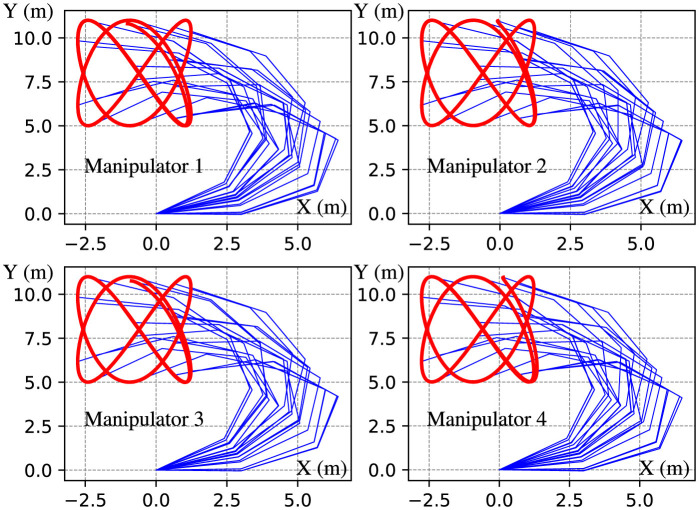
Trajectories of end effectors generated by DPB-ZNN algorithm (Equation 31) when robot manipulators track Lissajous pattern.

To control the redundant robot manipulators performing a cooperative control, for the entire system, a motion planning scheme for the entire system on the basis of the DTDTVCO problem is described as follows (Jin et al., 2018):


(41) $$\begin{array}{ll} \text{minimize } & \overset{4}{\underset{i=1}{\sum }}\frac{1}{2}{\dot{\theta }}_{i_{k}}^{\text{T}}{\dot{\theta }}_{i_{k}}, \end{array}$$

(42) $$\text{subject to }J_{iw_{k}}{\dot{\theta }}_{i_{k}}={\dot{r}}_{w_{k}},\;i=2,4,$$

(43) $$\begin{array}{l} \underset{j\in N_{i_{k}}}{\sum }||{\dot{\theta }}_{i_{k}}-{\dot{\theta }}_{j_{k}}|{|}_{2}=0,i=1,2,3,4, \end{array}$$

(44) $$\begin{array}{l} {\dot{\theta }}_{i}^{-}\leq {\dot{\theta }}_{i_{k}}\leq {\dot{\theta }}_{i}^{+},\;i=1,2,3,4. \end{array}$$

where ṙ_*i*_*w*__*k*__ denotes the time derivative of the desired trajectory of the end-effector position vector (i.e., reference), ${\dot{\theta }}_{i_{k}}\in {\mathbb{R}}^{n}$ denotes the joint velocities of the *i*th manipulator, and $J_{iw_{k}}\in {\mathbb{R}}^{2\times 5}$ denotes the Jacobian matrix of the manipulator *i*. In applications, limitations of joint velocities are often encountered. The boundary constraints are denoted as ${\dot{\theta }}_{i}^{-}\leq {\dot{\theta }}_{i}\leq {\dot{\theta }}_{i}^{+}$, where the lower and upper limits of the joint velocity ${\dot{\theta }}_{i}$ are denoted as ${\dot{\theta }}_{i}^{-}\in {\mathbb{R}}^{5}$ and ${\dot{\theta }}_{i}^{+}\in {\mathbb{R}}^{5}$, respectively. The bound constraints (Equation 44) are easily transformed into inequality constraints $J_{i1}{\dot{\theta }}_{i_{k}}\leq {\dot{\theta }}_{i}^{+}$ and $J_{i2}{\dot{\theta }}_{i_{k}}\leq -{\dot{\theta }}_{i}^{-}$, in which *J*_*i*1_ = *I*_*d*×*d*_ and *J*_*i*2_ = −*I*_*d*×*d*_, respectively. It is worth pointing out that, as discussed in Section 3.1, the equality constraints of the robot manipulators should not be contradictory. In this particular experiment, manipulators 2 and 4 share the same equality constraint $J_{iw_{k}}{\dot{\theta }}_{i_{k}}={ṙ}_{w_{k}}$, whereas manipulators 1 and 3 do not have such constraints. This means that manipulators 2 and 4 have access to the desired path, while manipulators 1 and 3 do not. Then, the DPB-ZNN algorithm (Equation 31) is adopted to solve the aforementioned cooperative control problem with parameters *h* = 0.2, σ_1_ = 50, and σ_2_ = 50. The sampling time is set to τ = 0.001 s. The desired trajectory for the robot manipulators follows a Lissajous pattern. In this simulation experiment, the upper and lower joint velocity limits for each joint were set to 1.5 and −1.5 rad/s, respectively. The experimental results are presented in Figure 8 through Figure 10.

**Figure 9:**
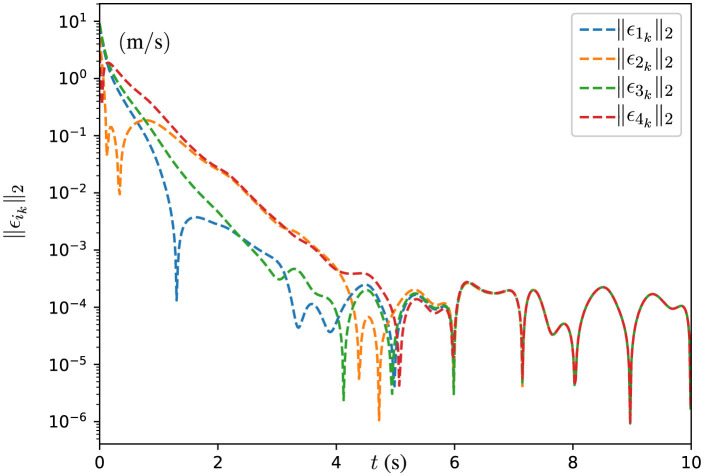
Trajectories of residual error ||ϵ_*i*_*k*__||_2_ of all robot manipulators.

The experimental results depicted in Figure 8 showcase the trajectories of the end effectors generated by the DPB-ZNN algorithm, as the manipulators track a Lissajous pattern. Each subplot, corresponding to Manipulator 1 through Manipulator 4, exhibits that all manipulators track the Lissajous pattern successfully. The experimental results depicted in Figure 9 reveal the effectiveness of the proposed DPB-ZNN algorithm in driving the residual errors $||\epsilon_{i_{k}}|{|}_{2}=||J_{iw_{k}}{\dot{\theta }}_{i_{k}}-{ṙ}_{w_{k}}|{|}_{2}$ of all robot manipulators toward zero over time. Figure 9 fully illustrates the efficiency of the proposed DPB-ZNN algorithm for solving the cooperative control problem of robot manipulators with joint velocity limits.

From Figure 9, one sees that the residual error trajectories, presented in a logarithmic scale reaches the level of 10^−4^ m/s to 10^−5^ m/s over a 10-s period, illustrate a rapid reduction in error magnitude, particularly during the initial phase of the simulation, highlighting the algorithm's swift convergence capabilities. While minor fluctuations in the early stages reflect the dynamic adjustments made by the algorithm to adhere to time-varying constraints and achieve consensus among agents, the overall trend consistently shows convergence across all manipulators. The slight variations in the error reduction rate among the manipulators are likely due to differences in their initial states and local objective functions, yet these discrepancies diminish over time, emphasizing the algorithm's distributed nature and ability to enforce consensus and feasibility. In addition, one sees that despite the lack of information of the desired path, manipulator 1 and manipulator 3 track the desired path successfully, meaning the cooperative control problem is solved by the DPB-ZNN algorithm (Equation 31) effectively in a fully distributed manner.

**Figure 10:**
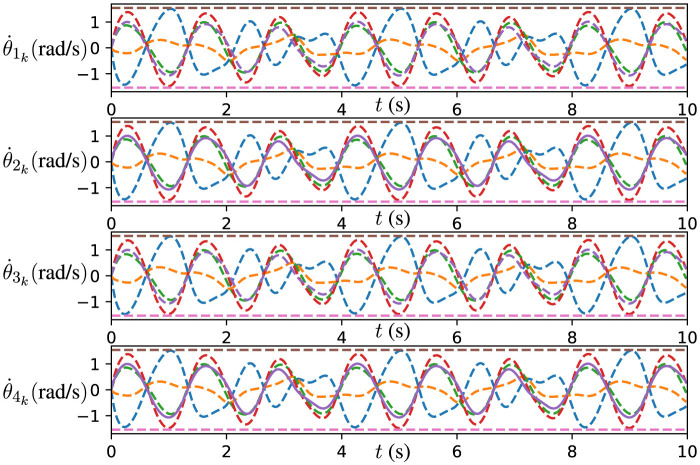
Trajectories of ${\dot{\theta }}_{i_{k}}$ for four robot manipulators generated by DPB-ZNN algorithm (Equation 31) when robot manipulators track Lissajous pattern.

Furthermore, the joint velocities of the robot manipulators solved by the DPB-ZNN algorithm (Equation 31) are presented in Figure 10. As shown in Figure 10, for all agents, all the joint velocities remain within the upper and lower joint velocity limits, which verify the effectiveness of DPB-ZNN algorithm in applying to the cooperative control problem of robot manipulators. To sum up, this experiment verifies the effectiveness and high accuracy of the proposed DPB-ZNN algorithm (Equation 31).

## 6 Conclusion

Aiming at solving the CTDTVCO problem with both time-varying equality and inequality constraints, a novel PB-ZNN model has been designed in this study by incorporating two penalty functions. The proposed PB-ZNN model solves the CTDTVCO problem in a semi-centralized manner. Then, on the basis of the PB-ZNN model, a DPB-ZNN algorithm has been proposed. By adopting an approximation formula, the DPB-ZNN algorithm solves the DTDTVCO problem in a fully distributed manner. The global convergence theorems of the proposed PB-ZNN model and DPB-ZNN algorithm have been proved. Numerical experiment results have illustrated the efficacy and efficiency of the proposed DPB-ZNN algorithm, including a simulation experiment applying it to the cooperative control of redundant robot manipulators.

## Data Availability

The original contributions presented in the study are included in the article/supplementary material, further inquiries can be directed to the corresponding author.
